# Is target sign (bull’s eye appearance) associated with adverse outcomes in COVID-19 patients? A case series and literature review

**DOI:** 10.22088/cjim.13.0.270

**Published:** 2022

**Authors:** Mohammad-Mehdi Mehrabi Nejad, Mohammadreza Salehi, Javid Azadbakht, Zahra Jahani, Parastoo Veisi, Nahid Sedighi, Sedighi Salahshour

**Affiliations:** 1 Department of Radiology, School of Medicine, Advanced Diagnostic and Interventional Radiology Research Center (ADIR), Imam Khomeini Hospital, Tehran University of Medical Sciences, Tehran, Iran; 2 Department of Infectious Diseases and Tropical Medicines, Tehran University of Medical Sciences, Tehran, Iran; 3 Department of Radiology, Faculty of Medicine, Kashan University of Medical Sciences, Kashan, Iran; 4 Brain and Spinal Cord Injury Research Center, Neuroscience Institute, Tehran University of Medical Sciences, Tehran, Iran; † Both authors contributed equally to this work

**Keywords:** Severe acute respiratory syndrome coronavirus 2, COVID-19, Tomography, Spiral computed, Pneumonia.

## Abstract

**Background:**

In COVID-19 pneumonia, chest CT scan plays a crucial role in diagnosing and closely monitoring lung parenchyma. The main reportedly chest CT features of novel coronavirus pneumonia (NCP) have been fully discussed in the literature, but there is still a paucity of reports on uncommon CT manifestations.

**Case presentation:**

Herewith, we have reported ten rRT-PCR confirmed COVID-19 patients with CT target signs (bull’s eye appearance); additionally, we have reviewed previously reported cases. Reviewing the literature, we found eight COVID-19 patients with target sign in the literature. 18 patients were included with a median age of 43. 11 (61%) patients were males. In 87% of patients, the lesions developed within the second-week post symptom onset. These patients mostly experienced an extended hospital stay (median = 10 days), with 53.8% of cases being admitted in ICU. The in-hospital mortality rate was 23%.

**Conclusion:**

Our findings indicate that lesions with a bull’s eye appearance are not significantly associated with higher mortality in hospitalized COVID-19 patients.

In mid-December 2019, novel coronavirus emerged in Wuhan, China, and then it quickly spread around China and throughout the world. Coronavirus disease 2019 (COVID-19) is a novel infectious disease that mainly causes inflammation in the respiratory system (1). Therefore, chest imaging is of diagnostic and monitoring value to detect and closely monitor lung parenchyma disease. The main reportedly chest CT features of novel coronavirus pneumonia (NCP) are multifocal, peripheral ground-glass opacity (GGO), consolidation, or a mixture of both with a basal dominancy, with or without less common findings such as the reverse halo sign (RHS) —a central area of GGO surrounded by a denser rim of consolidation— and crazy paving (2). Although typical clinical and para-clinical findings of NCP have been thoroughly investigated, there is still a paucity of reports on uncommon CT manifestations of NCP. Hereby, we present eight rRT-PCR confirmed COVID-19 patients with a target sign, a new unusual and yet specific tomographic sign in NCP; a literature review on formerly reported cases with this CT feature will be provided also.

## Case presentation

This retrospective observational study was approved by the local Institutional Review Board. The informed consent requirement was waived by the ethics committee of our institute (IR.TUMS.VCR.REC.1399.144). 

All on-admission chest CT scan of rRT-PCR confirmed COVID-19 infected patients, who were admitted to our tertiary referral hospital (with daily admission of about 15 COVID-19 patients) between February 2020 and February 2021 underwent review by an expert radiologist. Among them, all patients with opacities, suspicious for a target sign or bull’s eye appearance were included. 

Following data were obtained and enlisted for enrolled patients: (a) demographic information: age and sex; (b) on-admission oxygen saturation (SpO2); (c) pulmonary involvement (PI) score: each lobe was scored between 0-5 based on their involvement percentage (0: no involvement; 1: <5%; 2: 6-25%; 3: 26-50%, 4: 51-75%; and 5: >76%); the total score ranges from 0 to 25; (d) clinical data: hospital stay (days), ICU admission (and ICU length of stay in ICU admitted patients), mechanical ventilation, steroid therapy, calcium level, clinical outcome (death or discharge); and (e) time intervals (days): from admission to target appearance, and from symptom onset to target appearance.

Our systematic search was performed on relevant data through PubMed/MEDLINE and Google scholar. Articles published up to February 2021, using the search line of “target” or “target sign” or “bull’s eye” plus “COVID-19” or “novel coronavirus pneumonia” or “SARS-CoV2” were extracted. We included all studies enrolling rRT-PCR confirmed COVID-19 cases with bull’s eye appearance or target sign being reported on their chest CT scan. A total of four studies with eight relevant cases were found (table 1).

**Table 1:** detail of our studied patients and previously reported cases

	**age**	**sex**	**PI score**	**On-admission ** **SpO2**	**Hospital** Stay*	**ICU** **Admission**	ICU days*	**Mechanical ventilation**	**Steroid therapy**	Calcium**	**Admission to ** target appearance*	**Symptom onset to ** target appearance*	**Outcome**
Case 1	43	F	18/25	89%	12	No	0	No	Yes	7.1	10	18	Alive
Case 2	38	M	22/25	80%	35	Yes	20	Yes	Yes	8.6	5	14	Alive
Case 3	58	M	19/25	80%	7	Yes	7	Yes	Yes	-	0	6	Alive
Case 4	58	M	11/25	85%	7	No	0	No	Yes	8.1	0	10	Alive
Case 5	51	M	13/25	88%	10	Yes	3	Yes	Yes	8	7	-	Death
Case 6	66	M	18/25	89%	13	Yes	10	Yes	-	8.6	0	-	Death
Case 7	42	F	14/25	92%	13	Yes	5	Yes	Yes	7.7	7	-	Death
Case 8	42	F	11/25	93%	5	No	0	No	No	-	0	12	Alive
Case 9	56	M	10/25	90%	4	No	0	No	No	-	0	8	Alive
Case 10	54	M	15/25	87%	12	Yes	8	Yes	Yes	-	0	7	Alive
McLaren et al (14)	59 28	F F	patchy, bilateral, multifocal GGO	97% (with 2L O2) 91%	12 3	Yes No	At least 10 0	Yes No	No No	-	0 0	14 7	Alive Alive
Shaghaghi et al (13)	49	M	-	85%	5	No	0	No	No	-	0	4	Alive
Martin et al (11)	36 43 42	M F M	-	-	-	-	-	-	-	-	0-3	7-11	-
Muller et al (12)	37 38	F M	Mild/ moderately extensive parenchymal involvement	-	-	-	-	-	-	-	0	10-12	-
Total***	43	11/18 (61.1%) M	14.5/25	89%	10	7/13 (53.8%) Yes	3	7/13 (53.8%) Yes	7/12 (58.3%) Yes	8.05	13/18 (72.2%) 0	6<13/15 (86.6%)<14 (second week)	10/13 (76.9%) Alive

*: day **: mg/dL; normal range: 8.6-10.3 mg/dL ***: the most common record (n (%)) or median is reported.

PI: pulmonary involvement

ICU: intensive care unit

SO2: O2 saturation

M: male

F: female

Totally, 18 (10 from our study and 8 from previous studies) patients were included, detailed demographic and clinical data of which are summarized in table 1. 11 (61.1%) patients were males. The median age and SpO2 were 43 and 89%, respectively. Most patients had moderate-to-severe lung parenchymal involvement with median PI score of 14.5/25. Patients experienced extended hospital stay (median = 10 days) with 53.8% ICU admission rate and 23.1% in-hospital mortality. Patients with their calcium level being evaluated (8 out of 18), all showed hypocalcemia or calcium level on lower limit of normal range with median value of 8.05 mg/dL. Interestingly, 86.6% of patients developed target like opacities within the second week post symptom onset. 

**Figure 1:**
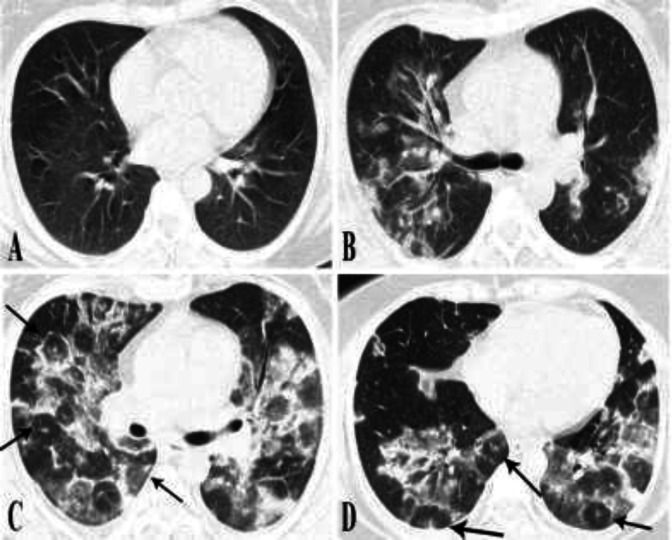
**Serial imaging of case 1.** The initial CT scan was normal (A). The on-admission CT scan taken after 8 days showed bilateral GGO with consolidative changes within (B). Ten days later, opacities increased in extension and density, with parenchymal band formation and several foci of target appearance (arrows) (C-D).

**Figure 2 (A, B):**
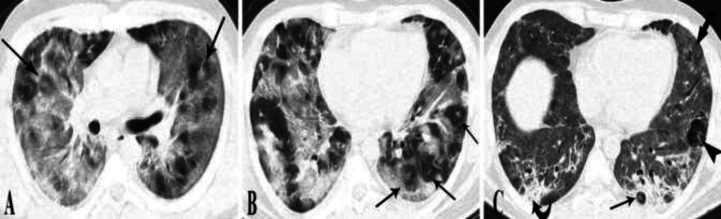
**Serial imaging of case 2.** The on-admission chest CT scan (A-B) depicts diffuse GGO and small foci of poorly developed target appearances (arrows). The second CT scan five days later shows diffuse residual faint GGO (thick arrow), parenchymal subpleural bands (curved arrow), and consolidating changes in both lower lobes. A few bullae (arrowhead) are also developed in the periphery of almost all pulmonary lobes. A small focus of target appearance is also present (arrow)

**Figure 3:**
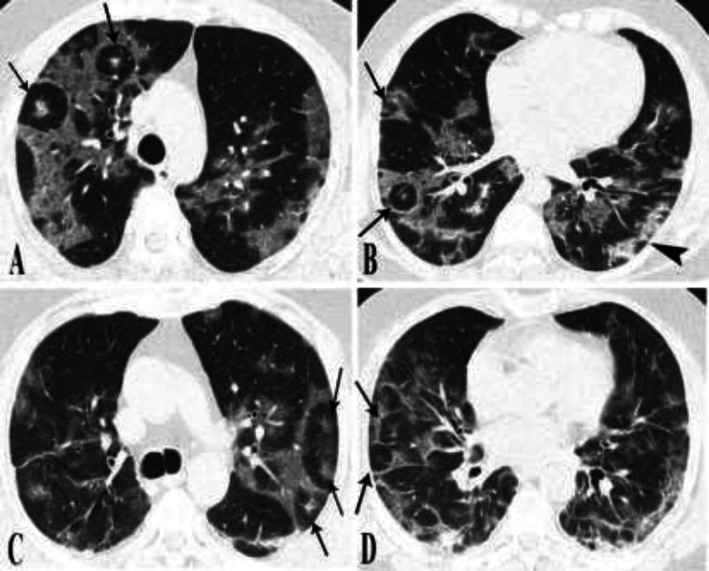
**Chest CT scans of cases 3-4. (A, B) case 3. **CT scan shows several foci of target shaped lesions (arrows). Arrowhead shows parenchymal band indicating the later phases of COVID-19 induced pneumonia. **(C, D)** **case 4.** On-admission Chest CT scan shows several foci of target appearance (arrows).

**Figure 4:**
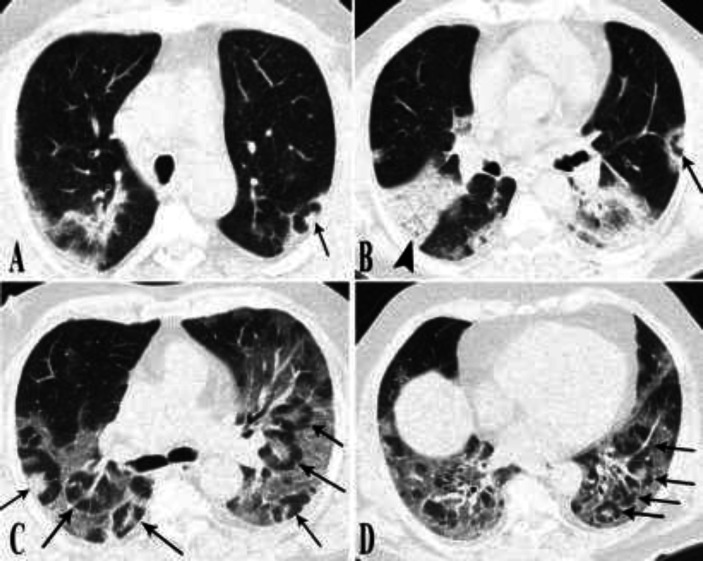
**Chest CT scans of cases 5-6.** **(A, B) case 5. **A few foci of target appearance were visible in the right lower and right upper lobes (arrows). Several foci of vacuolar sign imply the later phases of COVID-19 pneumonia (arrowhead).** (C, D) case 6.** Initial chest CT scan depicts several foci of target appearance in both upper and lower lobes bilaterally and in lingula (arrows). Few parenchymal bands are visible in favor of later phases COVID-19 pneumonia

**Figure 5:**
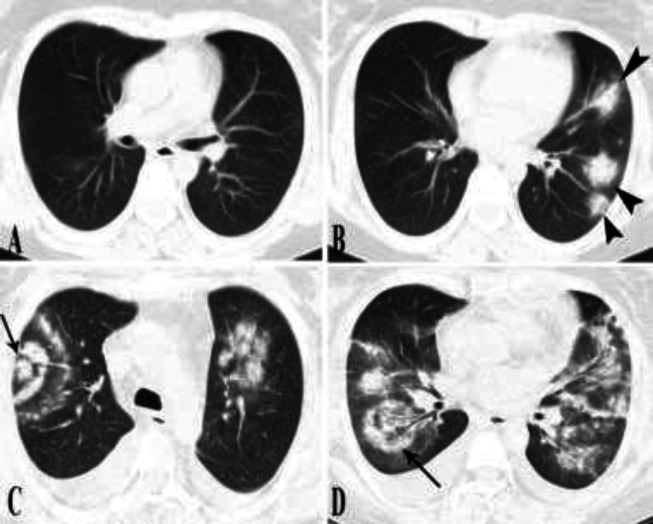
**Serial imaging of case 7.** Initial CT scan (A, B) depicts rounded consolidative opacities (arrowheads). One week later, two foci of target appearance developed in the right upper lobe and superior segment of right lower lobe (arrows). Mild cardiomegaly and bilateral pleural effusion are also present

**Figure 6:**
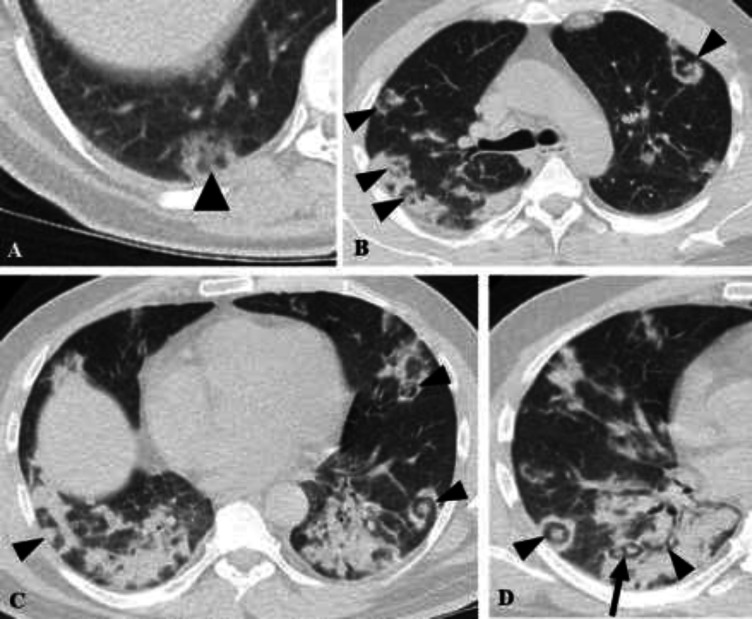
**Chest CT scans of cases 8-10. (A) Case 8.** CT scan shows a faint small center of denser opacity surrounded with a small area of GGO, which in turn is enclosed with a rim of consolidative opacity (arrowheads). (**B) Case 9. **Foci of target lesions indicated in both lungs are seen (arrowheads). (**C, D) Case 10.** Basal mixed density opacities with consolidation predominance containing air bronchogram are demonstrated. Multiple targetoid lesions are noted in both lungs (arrowheads). Arrow indicates two smaller target shaped lesions with some degrees of confluency

## Discussion

Looking into the archived chest CT scan of large series of hospitalized COVID-19 patients, we encountered ten cases with target lesions. Although this finding has been recently noticed and discussed in the literature, interestingly, most of these images were taken from March to November 2020 that waives the possibility of new variants of SARS-CoV-2 to be the causative pathogen. Pooling results from our study and previously reported cases, it appears that male sex and lower calcium level might increase the likelihood of target sign development in chest CT. Although COVID-19 prevalence is gender-independent, more extensive pulmonary involvement and more critical disease has been observed in infected males based on previous studies (3). Lower calcium level has also been found to be associated with severe disease and poor clinical outcome, as calcium is believed to have a role in cytokine release regulation (4, 5). 

Target sign mostly appears in the second week of symptom onset while there is moderate-to-severe pulmonary involvement. Parenchymal band and vacuolar sign were the most common simultaneous additional findings in CT images, indicating the later phases of COVID-19. According to the literature, pulmonary involvement begins with GGOs in the first stage (0-4 days), and is followed by crazy paving and consolidation in the second (5-8 days) and third (9-13 days) stages, respectively. Reportedly, pulmonary involvement peaks in the second week of disease course (6). 

Additionally, and of note, patients with this new tomographic sign stay in hospital for a longer period of time (median = 10 days) with a higher rate of ICU admission, aggressive treatment, and mechanical ventilation. The rate of ICU admission and in-hospital mortality in COVID-19 patients with target sign in chest CT were approximately 53.8% and 23%, respectively. On the other hand, the global statistics revealed that the rate of ICU admission and in-hospital mortality in COVID-19 patients are about 32% and 30%, respectively (7-9). Accordingly, although rare, patients with target-like opacities should receive intensive care and aggressive treatments to minimize the adverse events. However, the in-hospital mortality rate was not significantly higher than that of COVID-19 infected patients without targetoid lesion(s) in chest CT. 

Different COVID-19 radiologic manifestations in plain chest x-ray and CT scan have been fully discussed (10). According to the RSNA consensus, the most common typical CT manifestations of NCP are single/multiple peripheral bilateral patch (es) of GGO with or without consolidation, multifocal GGO of rounded morphology, crazy-paving, and RHS (2). Nevertheless, a growing number of case reports are offering atypical imaging features and specific signs in NCP. Some described radiology signs in COVID-19 pneumonia are white lung sign (diffuse opacities), halo sign (a nodule surrounded by GGO), RHS (rounded GGO surrounded by a ring of consolidation), bat wing sign (bilateral peripheral opacities), Rosa roxburghii sign (focal nodular GGO), and the gypsum sign (patchy bilateral consolidation with various density) (11). The target sign corresponds to a central rounded opacity surrounded by a polygonal ring-like opacity indicative of organizing pneumonia (12). So far, only eight cases of COVID-19 patients with target signs have been reported (11-14) (table 1). Although the appearance is distinctive, RHS and target signs could be misinterpreted in daily practice. The opacity outline is rounded or oval shaped in RHS, but polygonal in the target sign. The polygonal outline represents perilobular pattern, and is suggestive of organizing pneumonia (15); while the central nodular opacity indicates perivascular inflammation. Denser central opacities might at least partly reflect the focal enlargement of the pulmonary artery (16).Although RHS and target sign are relatively similar imaging manifestations and both indicate organizing pneumonia, target sign is more specific for COVID-19 (17). Patients with target sign should be carefully evaluated for COVID-19 in a suggestive clinical setting, as it should raise a flag to radiologists. Nevertheless, conducting large-scale investigations evaluating the prevalence of target sign in pneumonias caused by various pathogens are recommended to validate the specificity of this newly introduced tomographic sign in diagnosing COVID-19 induced pneumonia. Definitely drawing a conclusion from a limited population is subject to uncertainty and further studies using meta-analysis are warranted to confirm our findings.

In conclusion, in COVID-19 patients target opacities more likely develop in males and during second week after symptom onset. Patients with this specific chest CT feature experience an extended hospital stay and frequently require intensive care, but this sign is probably not associated with increased mortality rate. 
